# Influence Factors of Shot Peening Intensity and Its Effect on Residual Stress in Herringbone Gears

**DOI:** 10.3390/ma19214453

**Published:** 2026-10-23

**Authors:** Feng Yin, Liangliang Lv, Jinyuan Tang, Wen Shao

**Affiliations:** 1 State Key Laboratory of Precision Manufacturing for Extreme Service Performance, College of Mechanical and Electrical Engineering, Central South University, Changsha 410083, China; 2 Hunan Aviation Powerplant Research Institute, Aero Engine Corporation of China, Zhuzhou 412002, China

**Keywords:** shot peening intensity, herringbone gear, residual stress, shot peening

## Abstract

Shot peening intensity is a key parameter for evaluating process stability and the strengthening effect of shot peening. However, it is governed by the coupled effects of shot size, shot hardness, shot velocity and other process variables, and its relationship with the residual stress state of complex gear surfaces remains unclear. In this study, a residual stress to arc height calculation model for Almen strips was established and validated using dimple-size measurements and arc-height tests. The effects of S110, S170 and S230 cast steel shots, two shot hardness levels and different air pressures on shot peening intensity were then investigated. A sequentially coupled discrete element model—finite element model (DEM-FEM) method was further used to obtain the impact information and residual stress distribution on a herringbone gear tooth surface, and the effects of shot peening intensity on surface residual stress, maximum compressive residual stress, peak stress depth and compressive residual stress layer depth were analyzed. The results show that shot peening intensity increases with shot velocity, shot diameter and shot hardness. When the shot peening intensity increased from 0.158 mmA to 0.395 mmA, the depth of maximum compressive residual stress increased from 15 μm to 55 μm, and the compressive residual stress layer depth increased from 50 μm to 160 μm. In contrast, the surface residual stress and the magnitude of maximum compressive residual stress did not show a monotonic trend. The findings provide guidance for selecting shot peening parameters and evaluating the strengthening quality of herringbone gears.

## 1. Introduction

Herringbone gears use two helical gear sets with opposite helix directions to cancel axial force, while offering high load-carrying capacity, smooth transmission and a large contact ratio. They are therefore widely used in high-power-density transmission systems for aerospace, marine and heavy-duty equipment. However, gear roots and tooth flanks are subjected to alternating bending loads, contact loads and impact loads during service, and crack initiation, pitting and spalling remain major factors limiting their reliability and service life [1,2]. Recent studies on gear steels have shown that shot peening can reconstruct near-surface residual stress, hardness, roughness and microstructure, thereby significantly improving bending fatigue, contact fatigue and wear resistance [3,4,5].

During shot peening, numerous high-velocity shots repeatedly impact the material surface. Local elastoplastic deformation is constrained by the surrounding material, forming a gradient compressive residual stress field accompanied by work hardening, dislocation multiplication and grain refinement [6,7,8]. Compressive residual stress reduces the effective tensile stress under cyclic loading and delays crack initiation and propagation. Nevertheless, the strengthening effect does not increase monotonically with energy input; excessive pressure, coverage or peening time may increase surface roughness, induce local damage or promote residual stress relaxation [9,10,11]. Therefore, the evaluation of shot peening processes should consider surface residual stress, maximum compressive residual stress and its depth, and the compressive residual stress layer depth [12,13,14].

In engineering practice, shot peening intensity is usually characterized by the saturated arc height of an Almen strip. This index is convenient for controlling equipment and process stability, but it is essentially an integrated response governed by shot velocity, size, material, hardness, mass flow rate, incidence angle, stand-off distance and exposure time [15,16]. Previous studies have revealed the effects of air pressure and stand-off distance on shot velocity, the effects of shot size and coverage on residual stress and near-surface microstructure, and have established discrete-element/finite-element analysis methods based on Almen tests [17,18,19]. Because different parameter combinations may produce similar Almen intensities while the shot momentum, contact stiffness and number of impacts remain different, the residual stress state of a component surface cannot be uniquely determined from intensity alone [20,21].

To clarify these multiparameter coupling relationships, numerical studies of shot peening have evolved from regularly arranged few-shot models to random multi-shot models and coupled CFD-FEM and DEM-FEM models [15,17,18]. These methods can calculate impact velocity, position and impact number in a realistic or statistical sense and predict residual stress, surface morphology and work hardening [19,22,23]. Recent studies have further considered initial grinding stress, material strain-rate effects, crystal plasticity and impact-induced thermal effects [24,25,26]. These advances have improved prediction accuracy under complex conditions and also indicate that shot peening outcomes are jointly controlled by shot kinematics, material constitutive behavior and the initial surface state [27,28,29].

For complex curved components such as gears, shielding, collision and secondary impacts of shots in the tooth spaces change the velocity distribution and impact frequency at the tooth tip, pitch circle and tooth root [1,30]. Sequentially coupled DEM-FEM models have been used to predict the surface integrity of spur gears, spiral bevel gears and herringbone gears, and the results confirm that component geometry and nozzle motion alter local impact conditions [19,22,23]. Existing studies on herringbone gears have mainly discussed the strengthening uniformity at different tooth-surface positions. However, how equipment parameters first determine Almen intensity and how this intensity maps to the residual stress gradient of herringbone gears still require systematic quantitative clarification [24,28,31].

Existing studies also show that there is no single correspondence between shot peening intensity and residual stress characteristics. Increasing intensity generally drives the maximum compressive residual stress position and the compressive layer deeper into the material, whereas changes in surface stress and peak stress are jointly affected by shot size, hardness, coverage and material response [3,4,20]. Statistical optimization, analytical models and numerical models have been applied to process-parameter selection, but the relationship between shot type, hardness and velocity effects on Almen intensity and the residual stress of complex gear surfaces remains insufficiently understood [15,27,32]. This gap limits the assessment of the actual strengthening effect of herringbone gears when intensity is used as a quality-control index [33,34].

To address these issues, this study first establishes a residual-stress-to-arc-height calculation model for A-type Almen strips and validates the model using dimple-size and arc-height experiments. The effects of S110, S170 and S230 shots, two shot hardness levels and different shot velocities on shot peening intensity are then investigated. The purpose of this study is to explore the relationship between the peening strength and residual stress of the herringbone gears, as well as to investigate the influencing factors of peening strength. On this basis, a sequentially coupled DEM-FEM method for herringbone gears is used to obtain tooth-surface impact information and residual stress distributions. The relationships between shot peening intensity and surface residual stress, maximum compressive residual stress, peak-stress depth and compressive residual stress layer depth are quantitatively analyzed, providing a basis for selecting shot peening parameters and evaluating strengthening quality in herringbone gears.

## 2. Shot Peening Intensity Calculation Model

### 2.1. Model Establishment

**Figure 1:**
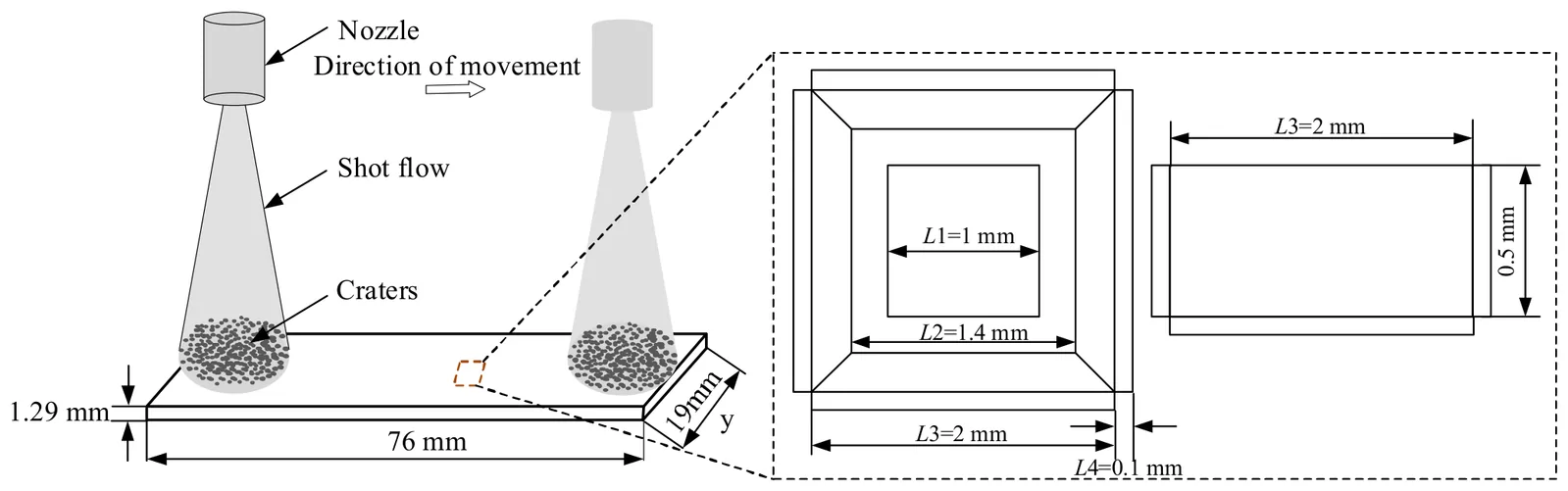
Geometry of the A-type Almen strip and residual stress calculation model.

An A-type Almen strip, which is commonly used in gear shot peening, was selected as the research object. Figure 1 shows the schematic diagram of Almen strip shot peening and the residual stress calculation model. The dimensions of the A-type Almen strip are 76 mm × 19 mm × 1.29 mm. A finite element model for shot peening was established using a local microscale surface unit to calculate the residual stress on the strip surface. The whole strip was then used as the research object, and the calculated residual stress was applied as an input condition to calculate the arc height caused by shot-peening-induced residual stress. The residual stress calculation model of the Almen strip has dimensions of 2 mm × 2 mm × 0.5 mm. Shots were generated in a 1 mm × 1 mm region at the center of the target, and Python (version 3.13) scripts were used to extract the residual stress results. The square region with side length L2 is the refined mesh region, where the element size is 10 μm × 10 μm × 10 μm. The region between L2 and L3 is the mesh transition region, where a progressive coarsening strategy was adopted; elements become coarser toward the target edge to reduce the total number of elements and improve computational efficiency without compromising accuracy. The region with side length L4 is the infinite-element region, where CIN3D8 elements were used to dissipate stress waves and improve calculation accuracy. All other regions were meshed using eight-node linear reduced-integration hexahedral elements (C3D8R).

**Figure 2:**
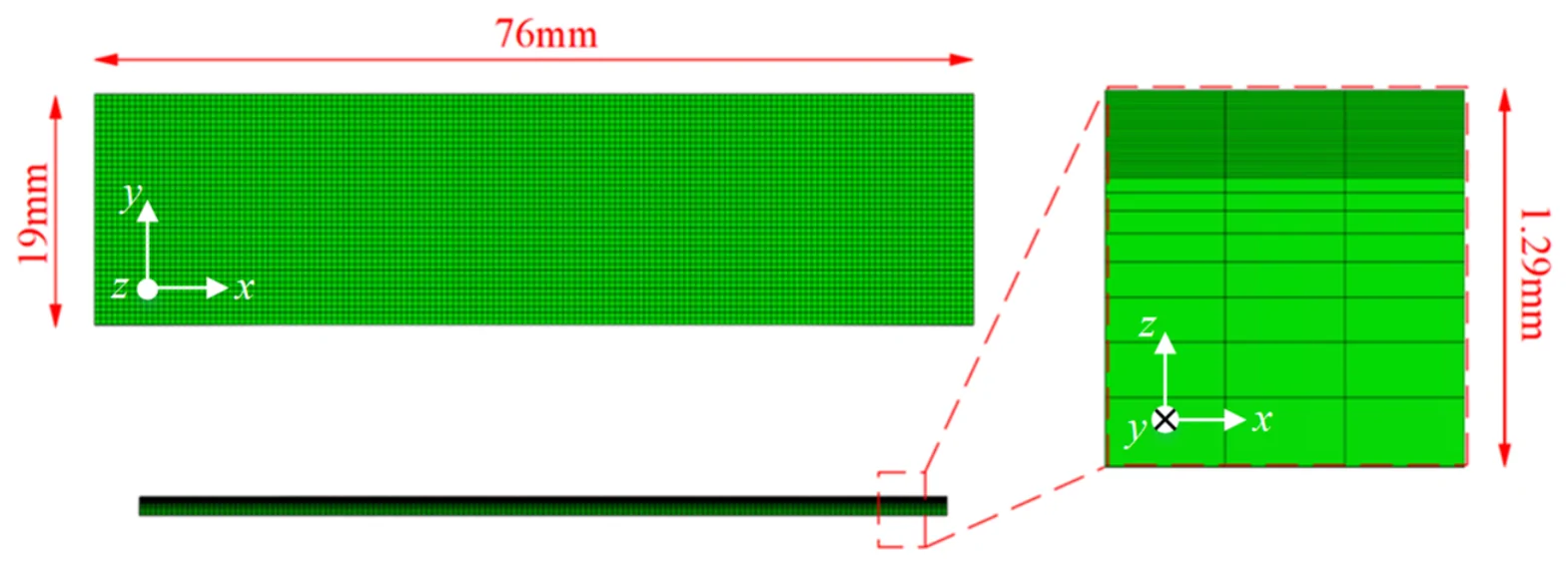
Geometry and mesh of the A-type Almen strip.

**Figure 3:**
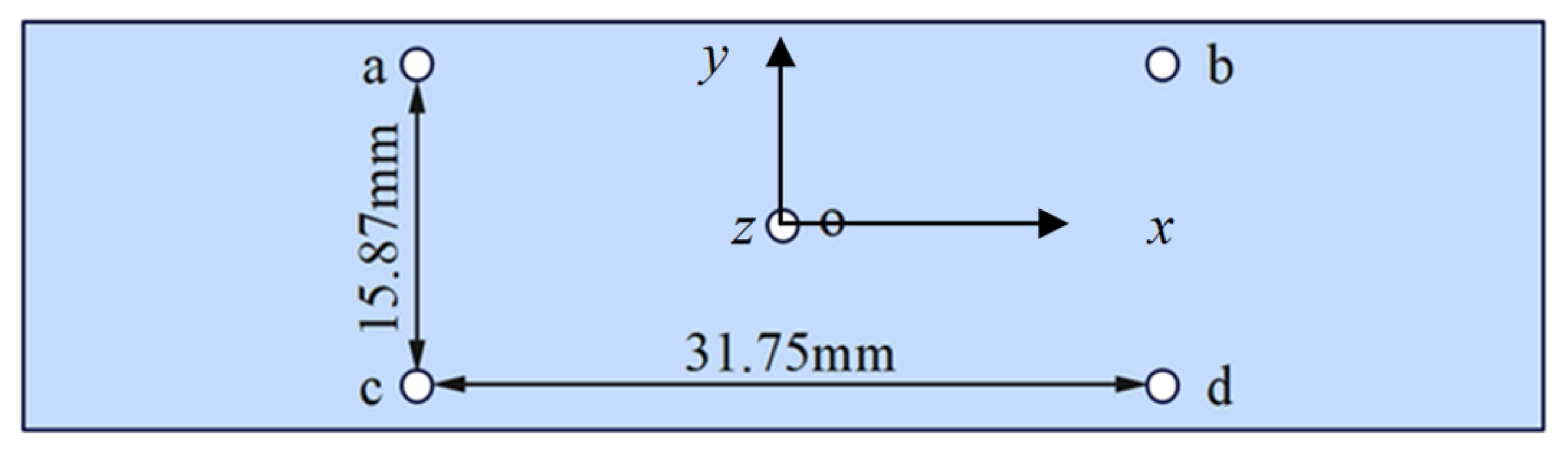
Measurement positions for arc height calculation.

After residual stress calculation, an arc-height calculation model for the Almen strip was established. The strip dimensions were 76 mm × 19 mm × 1.29 mm, and the mesh scheme is shown in Figure 2. Because the depth of compressive residual stress introduced by conventional shot peening usually does not exceed 0.3 mm, the mesh from the surface to a depth of 0.3 mm was uniformly divided with an element size of 10 μm × 10 μm × 10 μm. To improve computational efficiency, the region from 0.3 mm to 1.29 mm was meshed using a progressive coarsening strategy. The residual stress obtained from the model in Figure 1 was applied layer by layer along the depth direction to the elements in Figure 2, and the arc height was calculated using the Abaqus static solver. Following SAE J442, Test Strip, Holder and Gage for Shot Peening, and SAE J443, Procedures for Using Standard Shot Peening Test Strip, arc height was calculated from the height difference between the center point and the four corner points of a 31.75 mm × 15.87 mm rectangle at the strip center. As shown in Figure 3, the z-coordinates of points a, b, c, d and o were extracted; the differences between the z-coordinates of points a-d and point o were averaged to obtain the strip arc height.

Almen strips are typically made of 1070 spring steel, with a yield strength of 1238 MPa, Young’s modulus of 210 GPa, Poisson’s ratio of 0.3 and density of 7800 kg/m3. The shot types and parameters used in the simulations are listed in Table 1. Only S110 cast steel shot was used in the Almen strip peening experiments.

**Figure 4:**
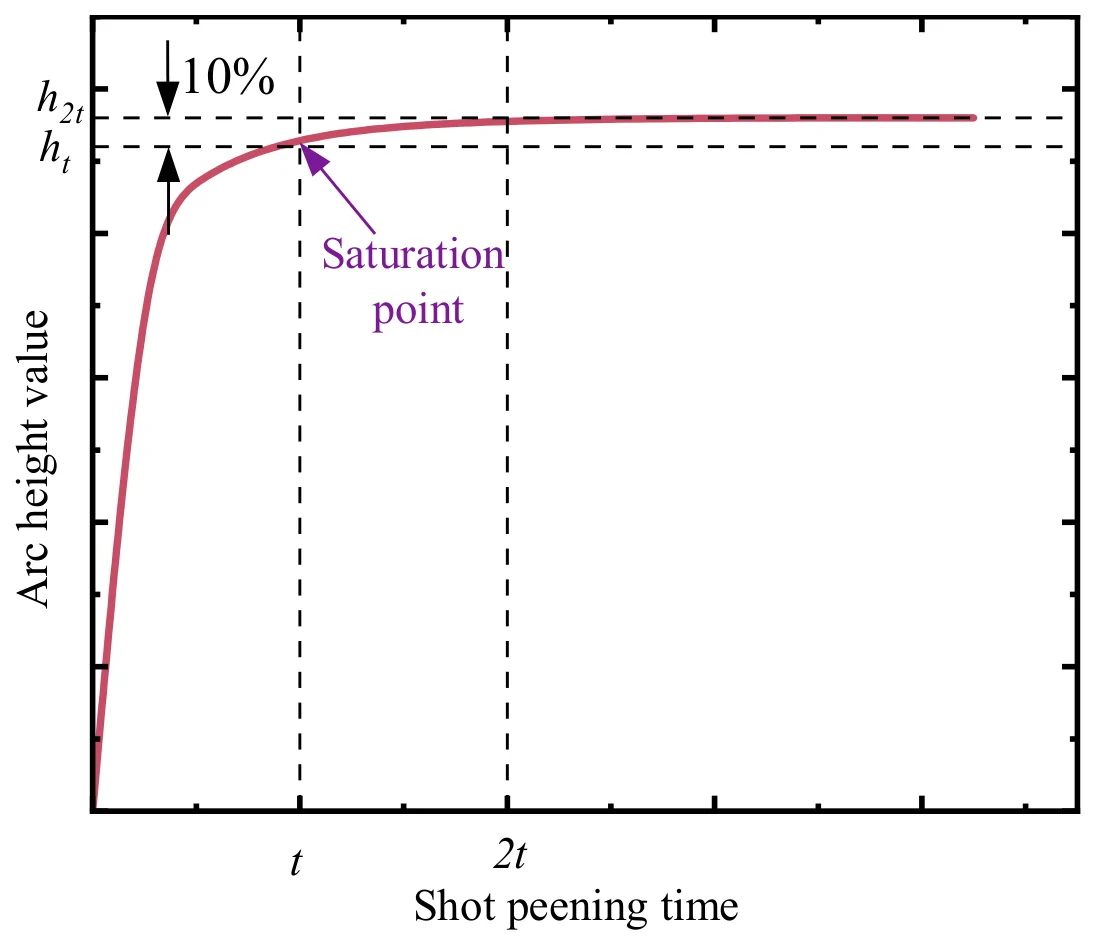
Saturation curve of shot peening intensity.

In the simulation, shot peening intensity was obtained from the saturated arc-height curve. The arc heights corresponding to different peening times were calculated and fitted to obtain the relationship between peening time and strip arc height, as shown in Figure 4. According to the definition of shot peening intensity, when the peening time increases from *t* to 2*t* and the strip arc height increases from *h_t_* to *h*_2*t*_, the condition *h*_2*t*_ − *h_t_* ≤ 0.1 *h_t_* must be satisfied. The arc height corresponding to time t is then defined as the shot peening intensity. Residual stresses under different peening times were calculated, the corresponding arc heights were obtained, and the arc-height curve was fitted to determine the shot peening intensity.

The gear material studied in this paper is 10CrNi2Mo3Cu2V steel. Before shot peening, the gear underwent carburizing, quenching, tempering, and precision grinding treatments. The yield strength of the gear material reached 1900 MPa, with the elastic modulus and Poisson’s ratio being 210 GPa and 0.3, respectively, and the density being 7800 kg/m^3^ [1]. 

### 2.2. Model Validation

In the experiment, the initial shot velocity was controlled by adjusting the nozzle air pressure. A high-speed camera was used to measure shot velocities under different air pressures [19] and these velocities were used as the initial shot velocities in the finite element calculation. The process parameters for Almen strip shot peening are listed in Table 2.

**Figure 5:**
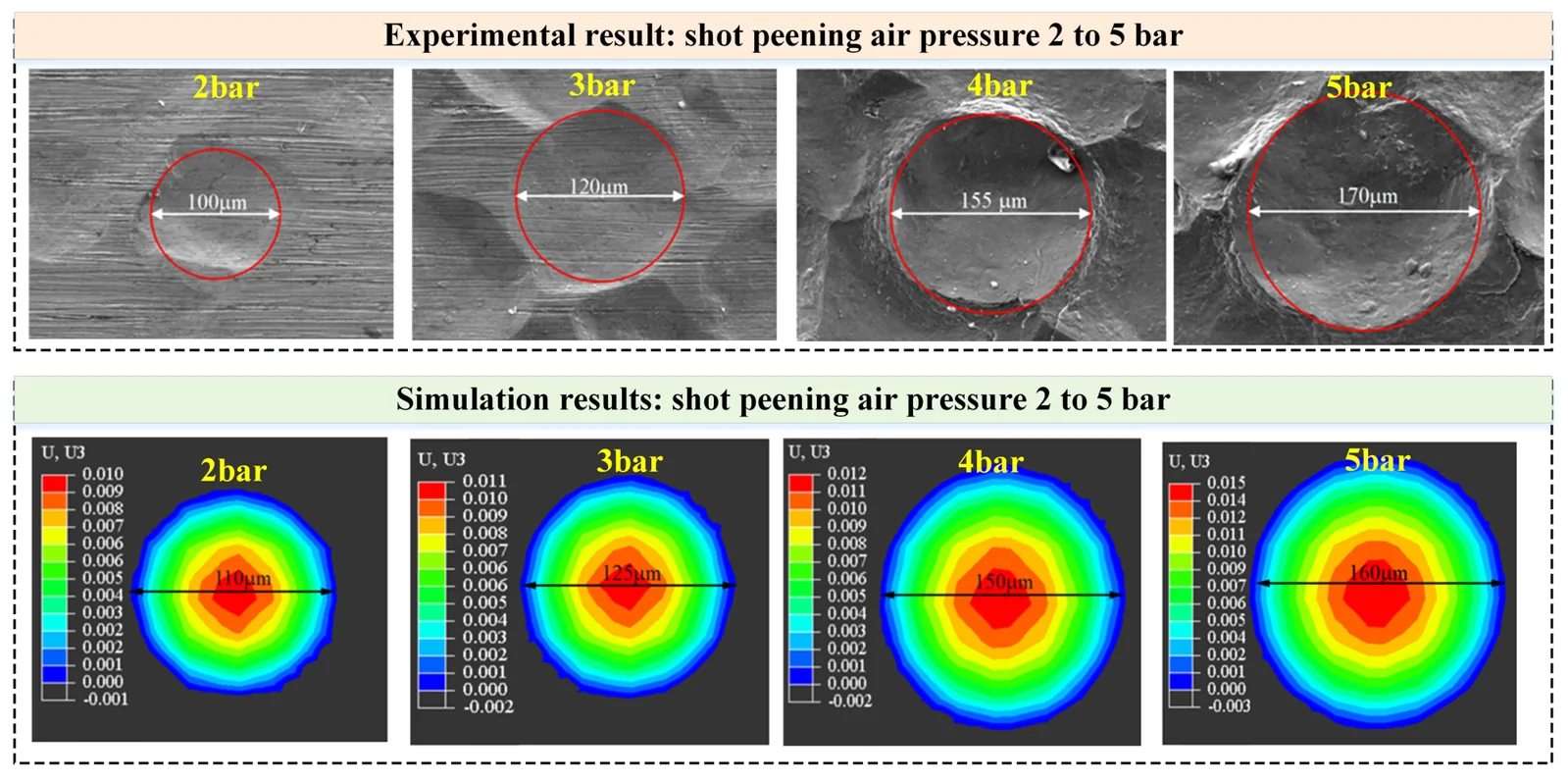
Experimentally measured and simulated dimple sizes.

Figure 5 shows SEM images of the Almen strip surfaces and simulated dimple sizes under different conditions. The dimple size increased continuously with increasing air pressure. In the experiment, when the peening air pressure increased from 2 bar to 5 bar, the dimple size increased from 100 μm to 170 μm. In the simulation, the dimple size increased from 110 μm to 160 μm over the same pressure range. At peening air pressures of 2, 3, 4 and 5 bar, the relative errors between the simulated and measured dimple sizes were 10%, 4.2%, 3.2% and 5.9%, respectively. These results demonstrate good agreement between the simulated and measured dimple sizes. It is worth noting that in the error statistics, the average value of at least 10 crater diameters was included. The average value of ten depressions under each set of shot peening parameters was taken as the current parameter’s average crater value, and the relative error between the simulation and the experiment was obtained.

**Figure 6:**
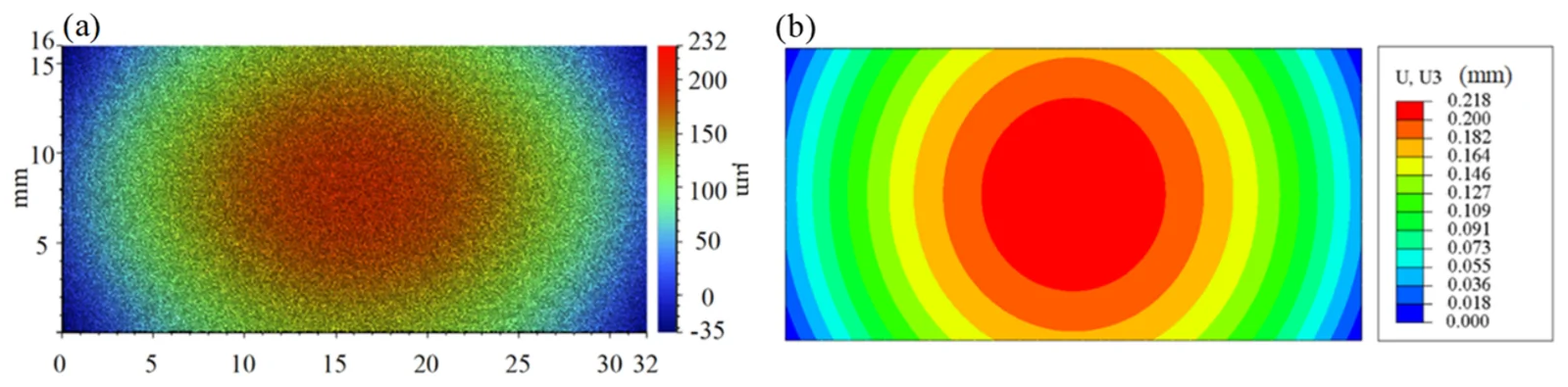
Measured and simulated Almen strip morphology at a peening air pressure of 3 bar: (**a**) Experimental results; (**b**) Simulation results.

Figure 6 shows the measured and numerically simulated morphologies of the Almen strip. The strip exhibited a symmetric profile with a higher center and lower sides, and the simulation agreed well with the experimental result. Figure 6 also shows the arc heights under peening air pressures of 3 bar and 5 bar. At 3 bar, the experimentally measured and simulated arc heights were 0.230 mmA and 0.218 mmA, respectively, corresponding to a relative error of 5.2%. Overall, the proposed method provides reliable accuracy in predicting shot peening intensity.

## 3. Residual Stress Calculation Model for Shot-Peened Herringbone Gears

**Figure 7:**
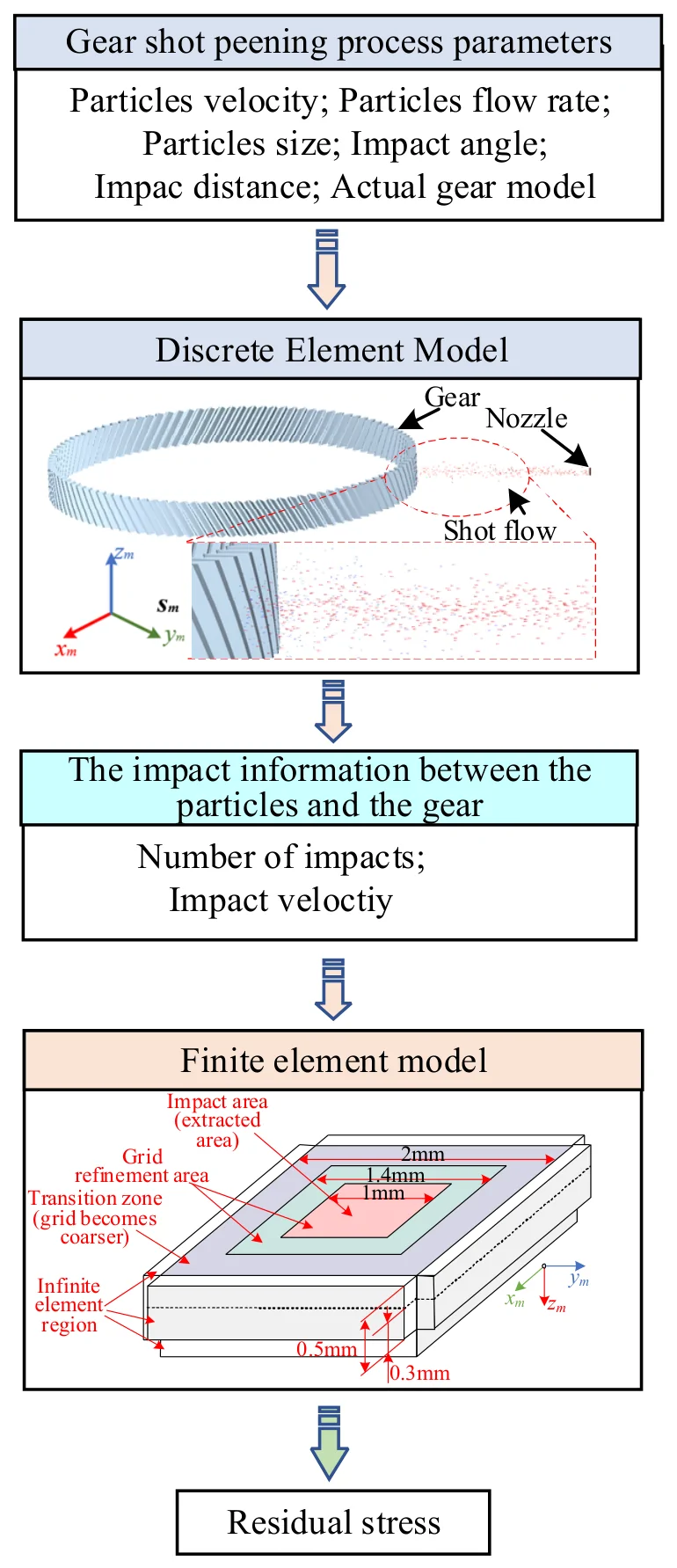
Calculation procedure for residual stress in herringbone gears.

The residual stress calculation model for herringbone gear shot peening consists of two parts: a DEM and a FEM. The DEM uses the whole gear as the research object to simulate the collision between the shot stream and tooth surface during shot peening, and finally obtains impact information at arbitrary tooth-surface positions, including impact velocity and impact number. The FEM uses a local tooth-surface region as the research object and calculates the residual stress at arbitrary tooth-surface positions using the impact information obtained from the DEM. The detailed calculation procedure is shown in Figure 7.

### 3.1. Discrete Element Model for Gear Shot Peening

#### 3.1.1. Discrete Element Geometric Model

**Figure 8:**
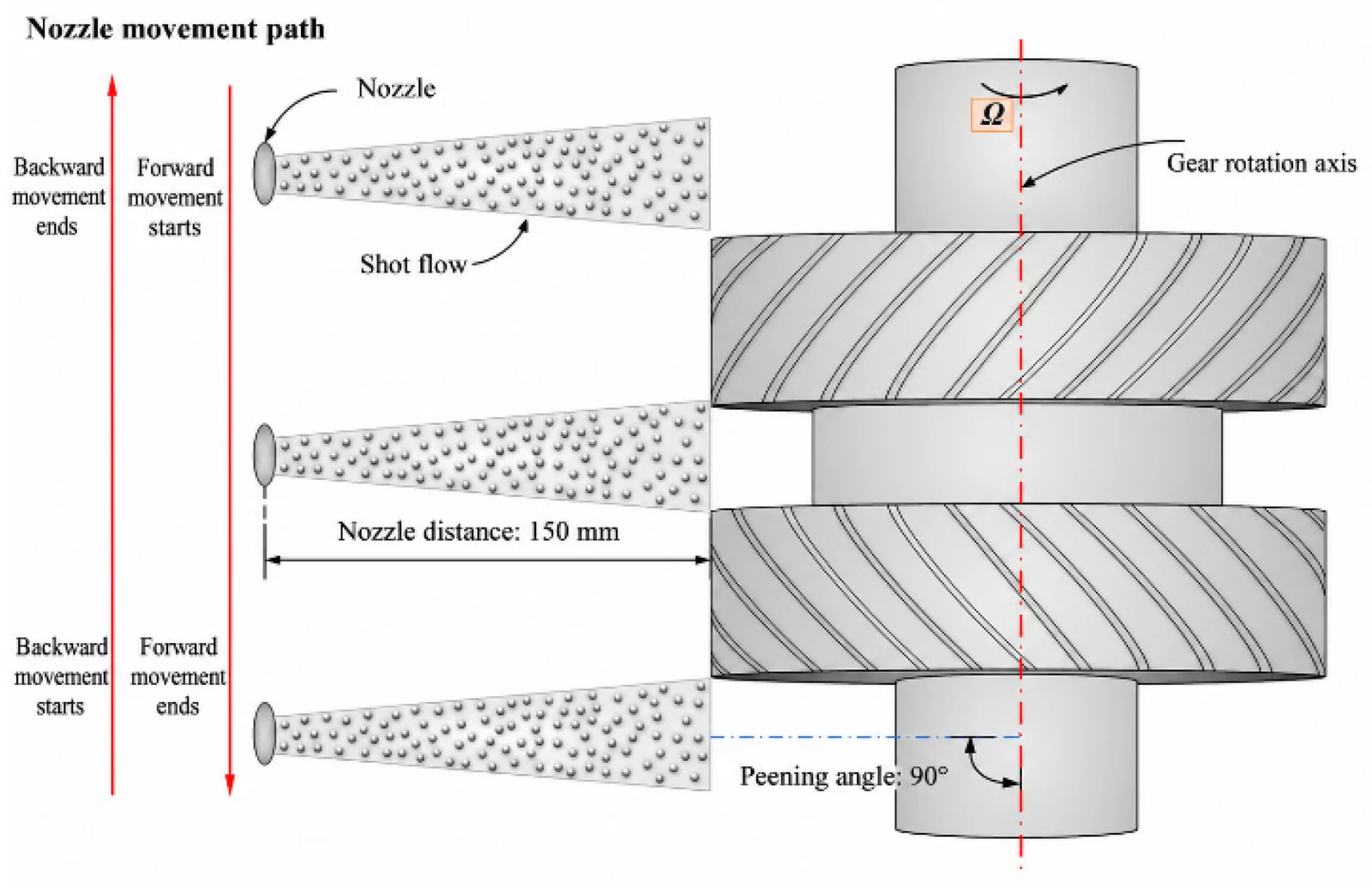
Schematic diagram of herringbone gear shot peening.

During herringbone gear shot peening, the nozzle is perpendicular to the tooth-tip direction and moves uniformly from one end of the gear to the other before returning, as schematically shown in Figure 8. If the complete herringbone gear tooth surface were directly constructed in the DEM simulation, the model size would be extremely large, resulting in substantially increased computation time. In addition, the central connecting shaft region of the herringbone gear would consume a large amount of ineffective computational resources.

To address this problem, two helical gear models with opposite helix directions were independently established to replace direct simulation of the complete herringbone gear. This simplification is valid because the tooth-profile equations of the left- and right-hand helical gears are identical.

#### 3.1.2. Basic Model Parameters

The following describes the detailed settings of the DEM model for herringbone gear shot peening. A Hertz-Mindlin no-slip contact model was used as the contact model in the DEM simulation. During modeling, shots were regarded as ideal smooth spheres, and the tooth surface was assumed to be smooth. The herringbone gear material was 10CrNi2Mo3Cu2V steel, and the shot material was cast steel. The material parameters of the shots and gear in the DEM model are listed in Table 3. The shear modulus was calculated from the elastic modulus and Poisson’s ratio.

**Figure 9:**
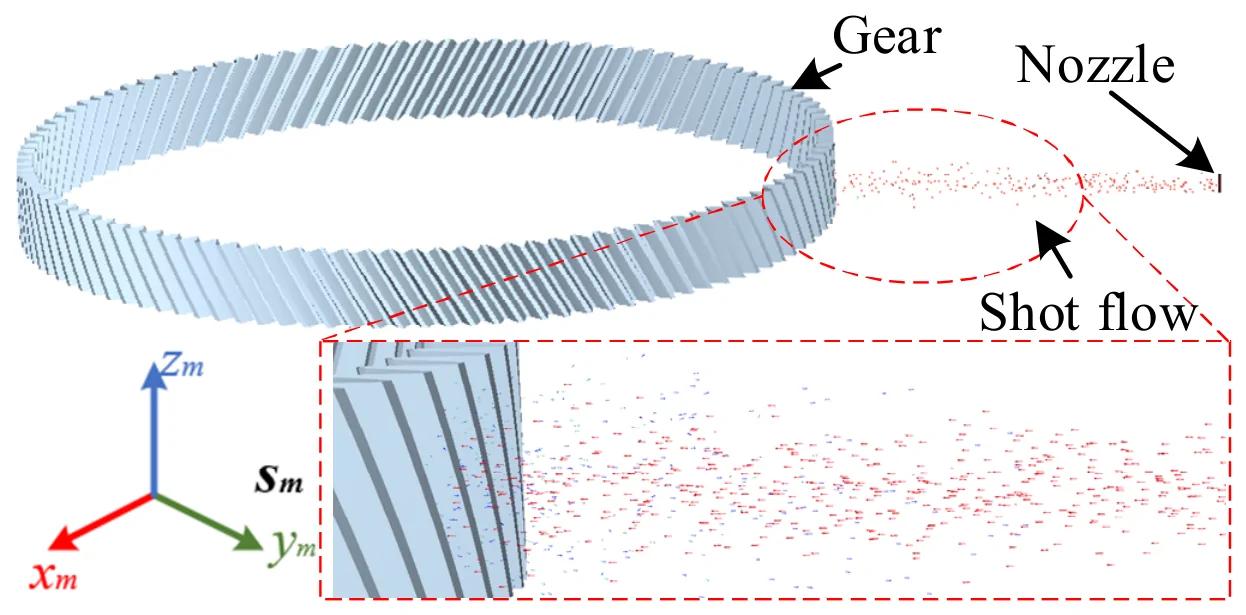
Geometry of the discrete element model for herringbone gear shot peening.

The solution domain of the DEM model should include the shot stream generated by the particle factory and the tooth-surface geometry, as shown in Figure 9. EDEM automatically divides the solution domain into cubic cells and performs collision detection in each cell during calculation. Therefore, fewer shots in each cell lead to higher collision-detection efficiency. If a cell contains only one shot or no shots, collision detection is unnecessary, which greatly improves computational efficiency. In general, the ideal cell size in a DEM model is twice the shot radius. The DEM time step Δtd is determined from the Rayleigh time step Δtr, calculated as follows [1]:

(1) $$\Delta t_{r}=\frac{\pi R_{s}{\left(\frac{\rho_{s}}{G_{s}}\right)}^{\frac{1}{2}}}{\left(0.1631\nu_{s}+0.8766\right)}$$

where *R*_s_ (mm) is the shot radius, *G*_s_ (GPa) is the shot shear modulus, and *ν_s_* is the shot Poisson’s ratio.

A smaller time step improves simulation accuracy. However, because a large number of shots are involved in shot peening and EDEM stores the calculation results for each time step, an excessively small time step consumes substantial computer memory. For DEM modeling, the time step Δtd is preferably selected as 20–40% of the Rayleigh time step Δtr. Therefore, Δtd was set to 30% of Δtr in this study.

### 3.2. Finite Element Model for Gear Shot Peening

#### 3.2.1. Geometric Model

**Figure 10:**
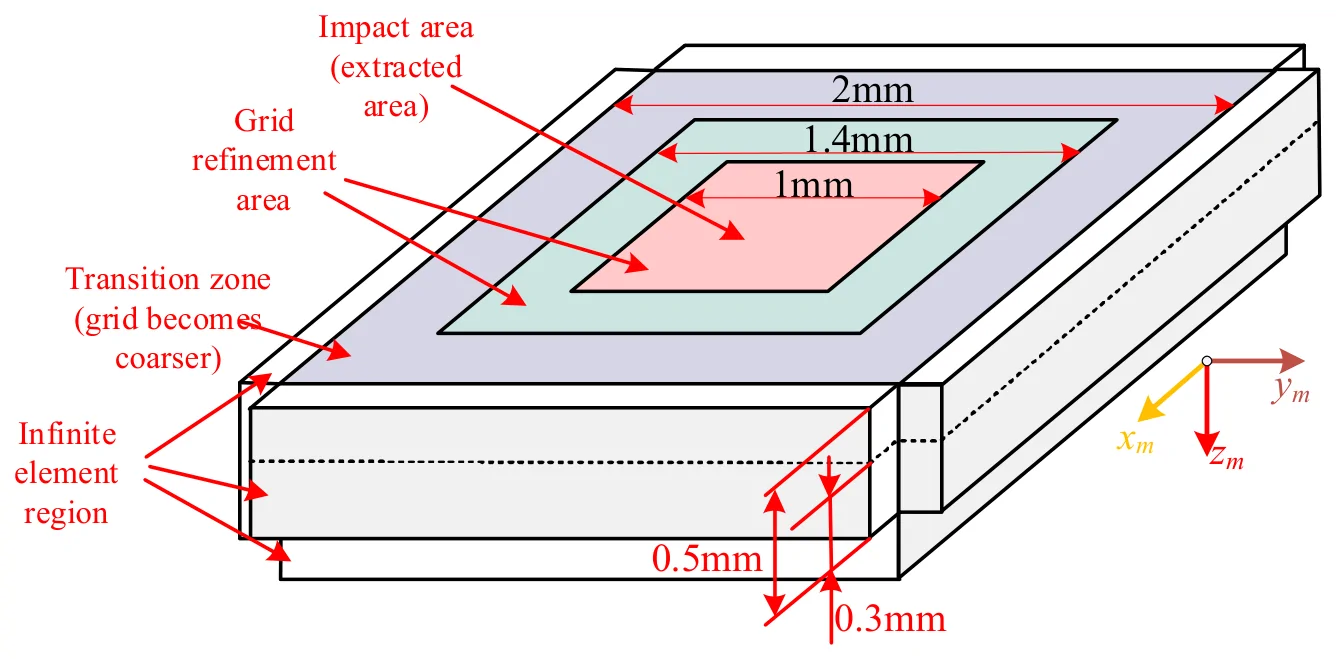
Geometry and mesh of the finite element model.

In the finite element model for herringbone gear shot peening, the target was defined as a cuboid with dimensions of 2 × 2 × 0.5 mm^3^, as shown in Figure 10. The central 1 × 1 mm^2^ region represents the local tooth-surface area where the peening result is calculated. The refined mesh region was set to 1.4 × 1.4 mm^2^, with an element side length of 10 μm. A 0.2 mm buffer region was reserved on each side, where the mesh size gradually increased. This meshing strategy reduces the number of elements in the non-core region as much as possible. In the depth direction (z direction), the first 0.2 mm was defined as the refined region, and the remaining 0.3 mm was meshed using a gradually increasing element size.

**Figure 11:**
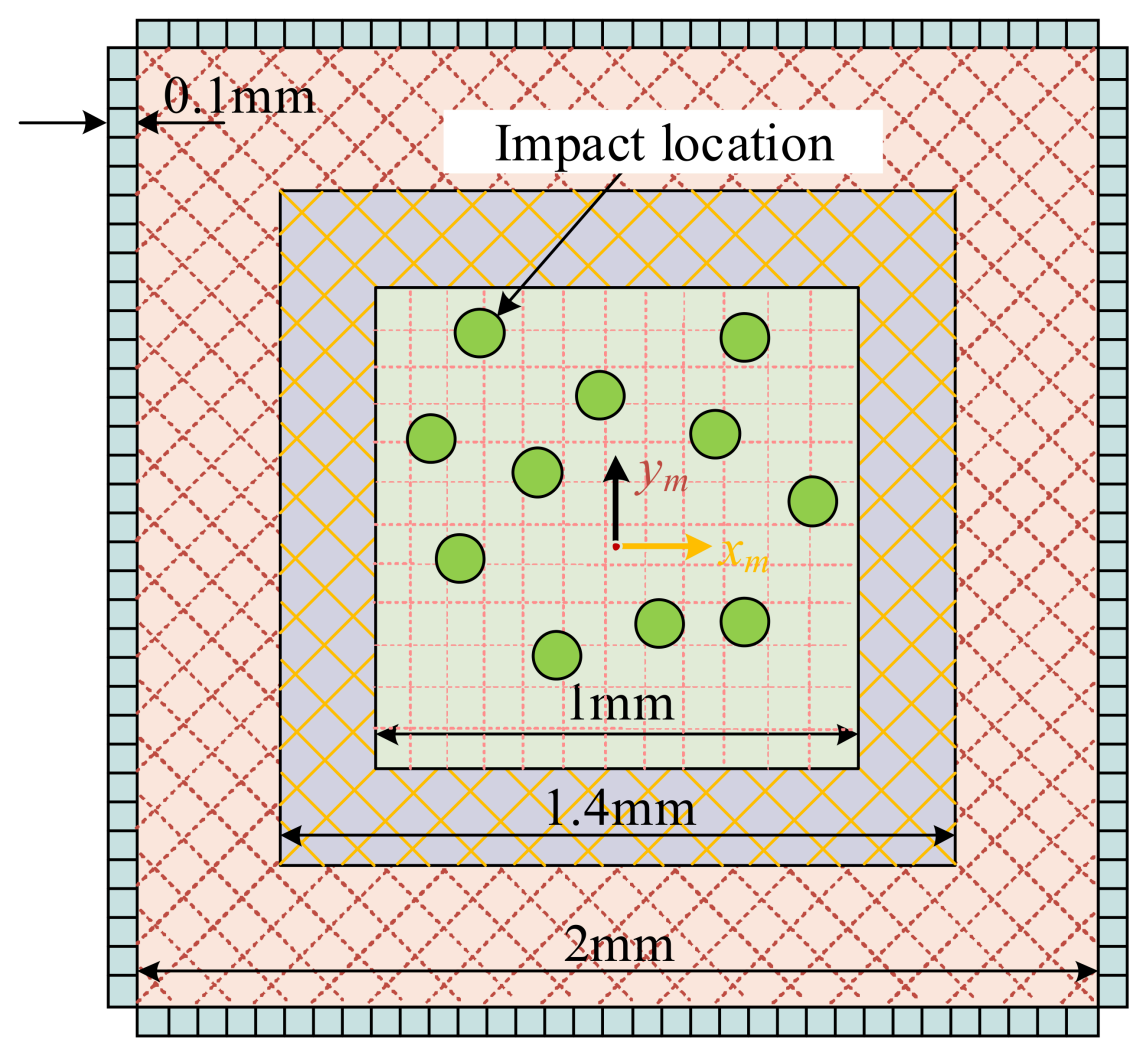
Mesh of the local tooth-surface region and schematic diagram of shot impact positions.

The refined and transition regions were meshed with C3D8R elements. In addition, as shown in Figure 10, a layer of semi-infinite elements (CIN3D8) was placed around the sides and bottom of the target. These semi-infinite elements were used to prevent stress waves from reflecting at the boundaries and superposing at the center, which would significantly reduce simulation accuracy. The shot diameters were set to 0.35 mm (S110), 0.5 mm (S170), and 0.7 mm (S230), and the shots were meshed using C3D8R elements. The particles randomly impact on the surface of the target plate at positions as shown in Figure 11.

#### 3.2.2. Constitutive Model

After defining the geometry and mesh, the mechanical behavior and boundary conditions of the material were specified to ensure accurate simulation of the shot impact process. The gear material investigated in this study was 10CrNi2Mo3Cu2V steel, and its chemical composition is listed in Table 4.

The Johnson-Cook (J-C) model was used to describe the mechanical behavior of the material under repeated impacts during shot peening. The expression is [25]:

(2) $$\sigma_{y}=(A+B\varepsilon_{p}^{n})\left(1+C\ln\frac{\varepsilon_{p}^{\prime }}{\varepsilon_{0}}\right){\left(1-\frac{T-T_{r}}{T_{m}-T_{r}}\right)}^{m}$$

where *σ_y_* is the yield stress, *ε_p_* is the equivalent plastic strain, $\varepsilon_{p}^{\prime }$ is the equivalent strain rate, *ε*_0_ is the reference strain rate, *T* is the test temperature and *T_r_* is the reference room temperature. *A*, *B*, *n*, *C* and *m* are material constants. A represents the initial yield strength under quasi-static conditions, *B* is the strain-hardening coefficient, n is the strain-hardening exponent, *C* is the strain-rate sensitivity coefficient, and *m* is the thermal softening coefficient. The three terms on the right-hand side represent static hardening, strain-rate strengthening and thermal softening, respectively. The values of the five constants are listed in Table 5 [1].

In the finite element model, the elastic moduli of the shots and target were both set to 210 GPa, Poisson’s ratios to 0.3, and densities to 7800 kg/m^3^. The mechanical properties of the target strictly followed the J-C constitutive model described above.

The shots were simplified as smooth spheres, and an isotropic hardening model was used to describe shot hardening during peening. Because direct measurement of shot mechanical properties is technically difficult, and mature approaches exist in the literature for converting shot hardness into mechanical parameters in finite element simulations of shot peening, this study adopted the same strategy. The initial yield strength of the shot was calculated using the following empirical equation [1]:

*σ_ys_* = 2.876*HV* − 90.7 (3)

where *σ_ys_* is the shot yield strength and *HV* is the Vickers hardness. The hardness of high-hardness cast steel shots ranges from 55 to 62 HRC, corresponding to 595–746 HV. The average value of 670 HV was used to calculate the shot yield strength.

#### 3.2.3. Damping Parameters

The shot peening FEM model was solved using the explicit dynamics module in Abaqus. Even after a shot impact is completed, the z-direction displacement and other physical quantities of the target surface nodes continue to oscillate around the equilibrium position. To maintain high computational accuracy, the next shot should impact only after the displacement oscillation of the target surface has gradually stabilized, which greatly increases computation time. To reduce stabilization time, Mahmoudi et al. [23] introduced Rayleigh damping into the target material model. Rayleigh damping is a combination of the mass and stiffness matrices and can be expressed as:

***C*** = *α****M*** + *β****K*** (4)

where ***C*** is the damping matrix, *α* is the mass-proportional damping coefficient, ***M*** is the element mass matrix, *β* is the stiffness-proportional damping coefficient, and ***K*** is the element stiffness matrix. Studies have shown that when *α* and *β* are 6 × 10^6^ and 0, respectively, the displacement oscillation at the center node of the target surface is significantly reduced [35].

For the boundary conditions of the finite element model, the bottom surface of the target was fully fixed. The contact between the shot and target was defined as surface-to-surface contact, with hard contact in the normal direction and penalty friction in the tangential direction. Because the friction coefficient (0.1 < f < 0.5) has little influence on plastic strain [23], it was set to 0.2 in this study [36].

#### 3.2.4. Grouped Calculation Strategy

The established DEM model for herringbone gear shot peening can calculate impact information at any local tooth-surface position of interest. These DEM results can then be used as inputs for the shot peening FEM model to calculate residual stress distributions and surface morphology evolution. The impact velocity of the *i*-th shot at a local tooth-surface position is defined as:

(5) $$v_{i}={\left[v_{x}^{i},v_{y}^{i},v_{z}^{i}\right]}^{T}$$

Previous studies have shown that shot impact positions on the tooth surface are continuously and uniformly distributed. The impact position of the *i*-th shot in the local tooth-surface region is expressed as:

(6) $$P_{i}={\left[P_{x}^{i},P_{y}^{i},P_{z}^{i}\right]}^{T}$$

where $P_{x}^{i}$ and $P_{y}^{i}$ follow uniform distributions, expressed mathematically as:

(7) $$\left\{\begin{array}{l} P_{x}^{i}\sim U\left(-0.5,0.5\right)\\ P_{y}^{i}\sim U\left(-0.5,0.5\right)\\ P_{z}^{i}=0 \end{array}\right.$$

In an actual shot peening process, however, collisions between shots and the tooth surface occur at time intervals rather than simultaneously. To simulate this process more accurately, the initial positions of the shots in the FEM model must be considered so that the shots impact the target sequentially. The initial position Pc(i) of the *i*-th shot is expressed as:

(8) $$P_{c}\left(i\right)=P_{i}-v_{i}\times i\times t_{\mathrm{FEM}}-{\left[0,0,\frac{R_{s}}{2}\right]}^{T}$$

where *t*_FEM_ is the time interval between two consecutive shot impacts at a local tooth-surface position. To ensure that the displacement of the target surface nodes reaches a stable state before the next shot impact, *t*_FEM_ was set to 10^−6^ s [1].

During gear shot peening, the number of shot impacts per unit area on a local tooth-surface region can reach several hundred to several thousand. If all shots are included simultaneously in a single finite element model, the required computational resources become excessive and the calculation time becomes unacceptable. To overcome this problem, the Import function in Abaqus was used to implement a grouped import calculation strategy. The basic idea is to decompose the large number of shot impacts into multiple continuous sub-analyses. First, according to the impact number per unit area, *C*_psm_, shots were divided into *K* groups with 10 shots in each group. The first group analysis used the ground tooth-surface state, including the initial residual stress distribution and surface morphology, as the input condition. Abaqus stores the results of each analysis in an odb file. Starting from the second group, a chain calculation mode was used in which the odb file from the previous analysis was imported as the initial state for the next group. This strategy enables efficient calculation of residual stress and surface morphology evolution under a large number of impacts.

## 4. Gear Shot Peening Experiment and Model Validation

**Figure 12:**
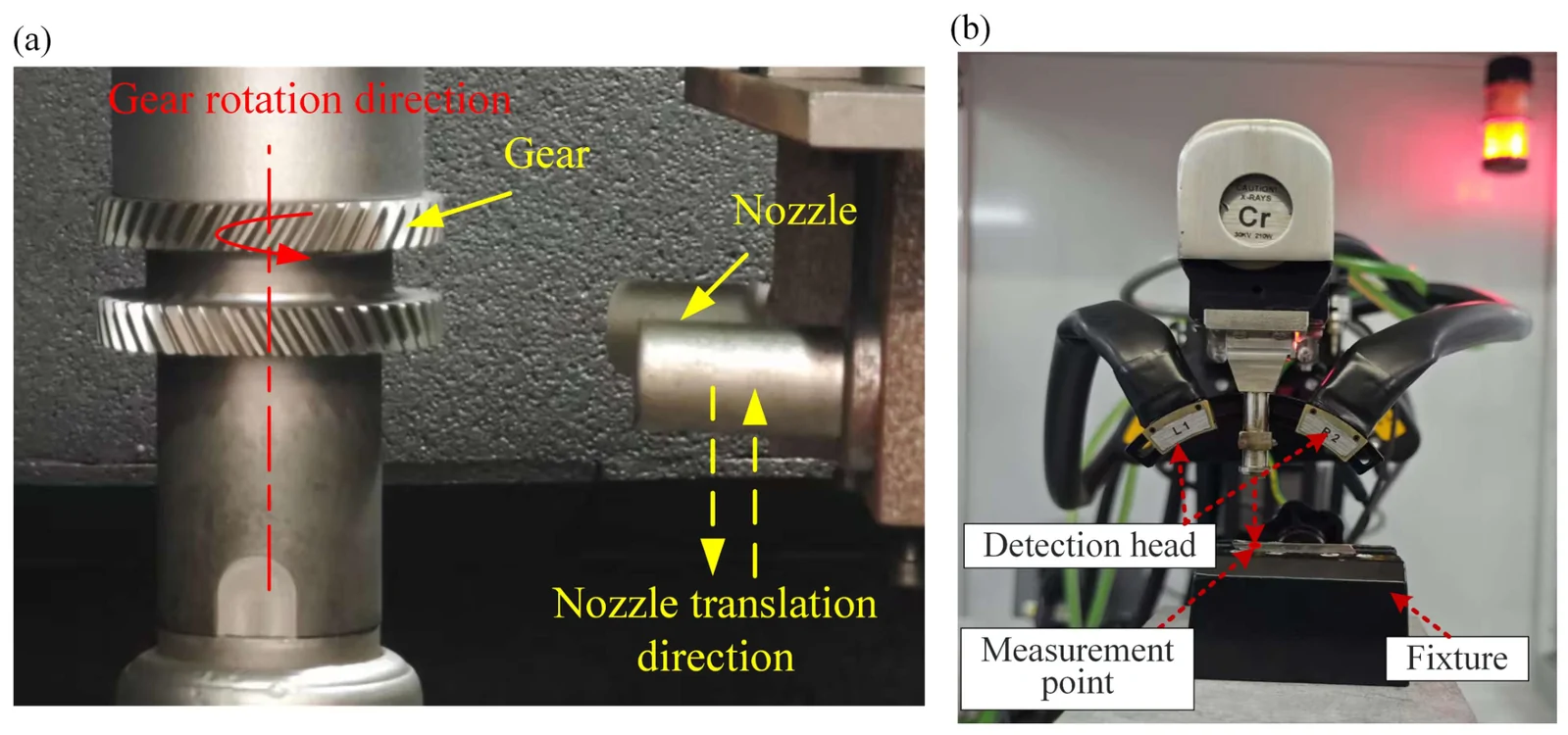
(**a**) Herringbone gear shot peening experiment. (**b**) Residual stress testing process.

**Figure 13:**
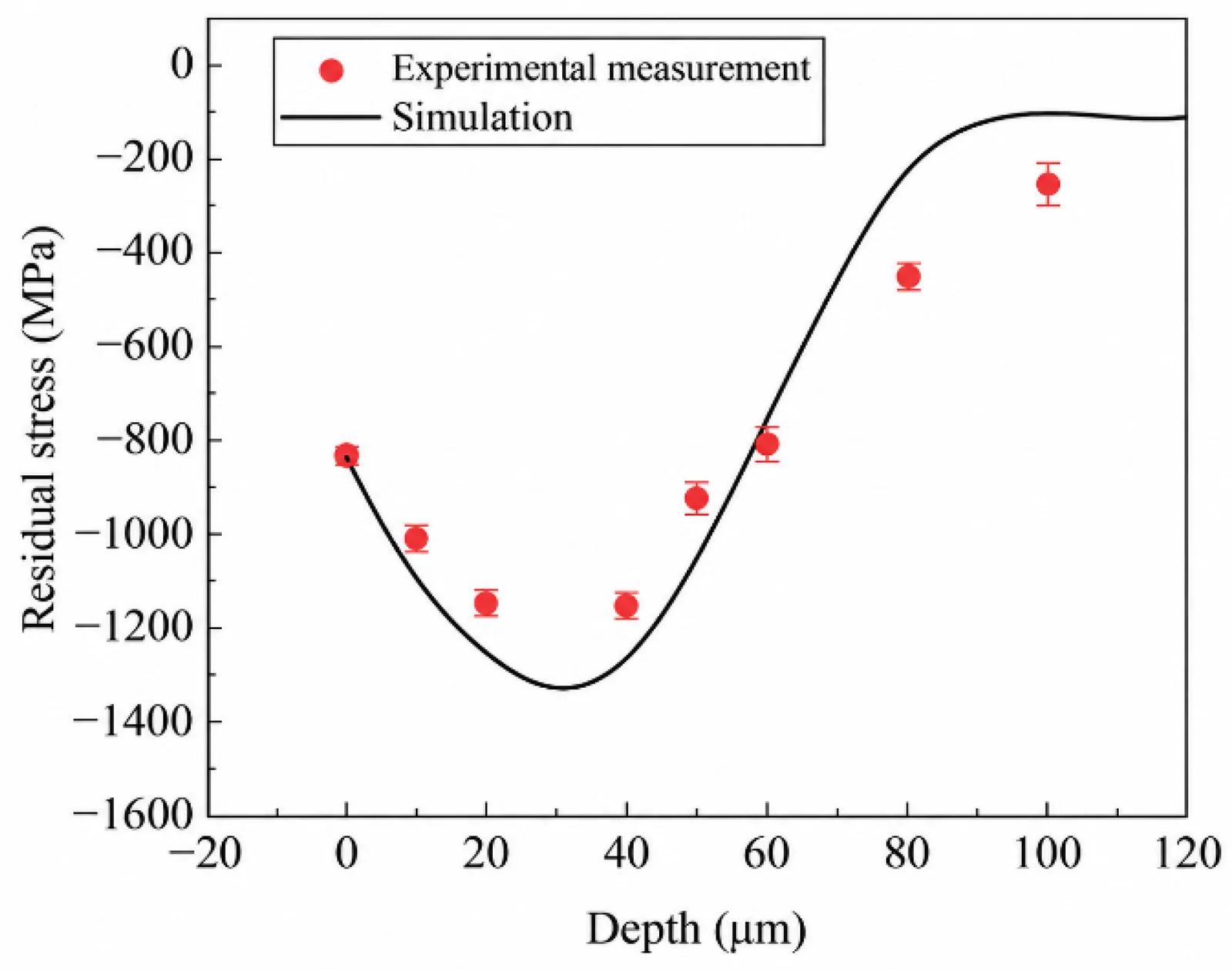
Comparison between measured and simulated residual stress [1].

Figure 12a shows the herringbone gear shot peening experiment. The peening coverage was 200%; S110 high-hardness cast steel shot was used; the shot mass flow rate was 6 kg/min, the gear rotational speed was 20 rpm, and the peening air pressure was 2.5 bar. Figure 13 shows the residual stress at different depths. Both the measured and simulated surface residual stresses were approximately −830 MPa, and the maximum residual stress appeared at a depth of 30 μm. The measured maximum residual stress was −1250 MPa, while the simulated value was −1350 MPa, corresponding to a relative error of 8%.

The residual stress measurement equipment uses an X-ray diffraction instrument (iXRD 300 W) produced by Proto Company in LaSalle, Ontario, Canada, as shown in Figure 12b. This equipment uses the X-ray diffraction method to conduct non-destructive tensile stress and shear stress measurements on polycrystalline metal material components. During the test, the X-ray target material is a Cr target, and the test crystal plane is selected as (211). The test conditions are: the tube voltage of the equipment is 25 kV, the tube current is 5 mA, and the diameter of the collimator is 1 mm. The voltage is 25 kV, the current is 5 mA, the diameter of the collimator aperture is 1 mm, the exposure time is set to 3 s, and the exposure times are set to 7 times. During the measurement, the maximum swing Beta angle of the X-ray tube is 20°.

## 5. Results and Discussion

### 5.1. Factors Influencing Shot Peening Intensity

**Figure 14:**
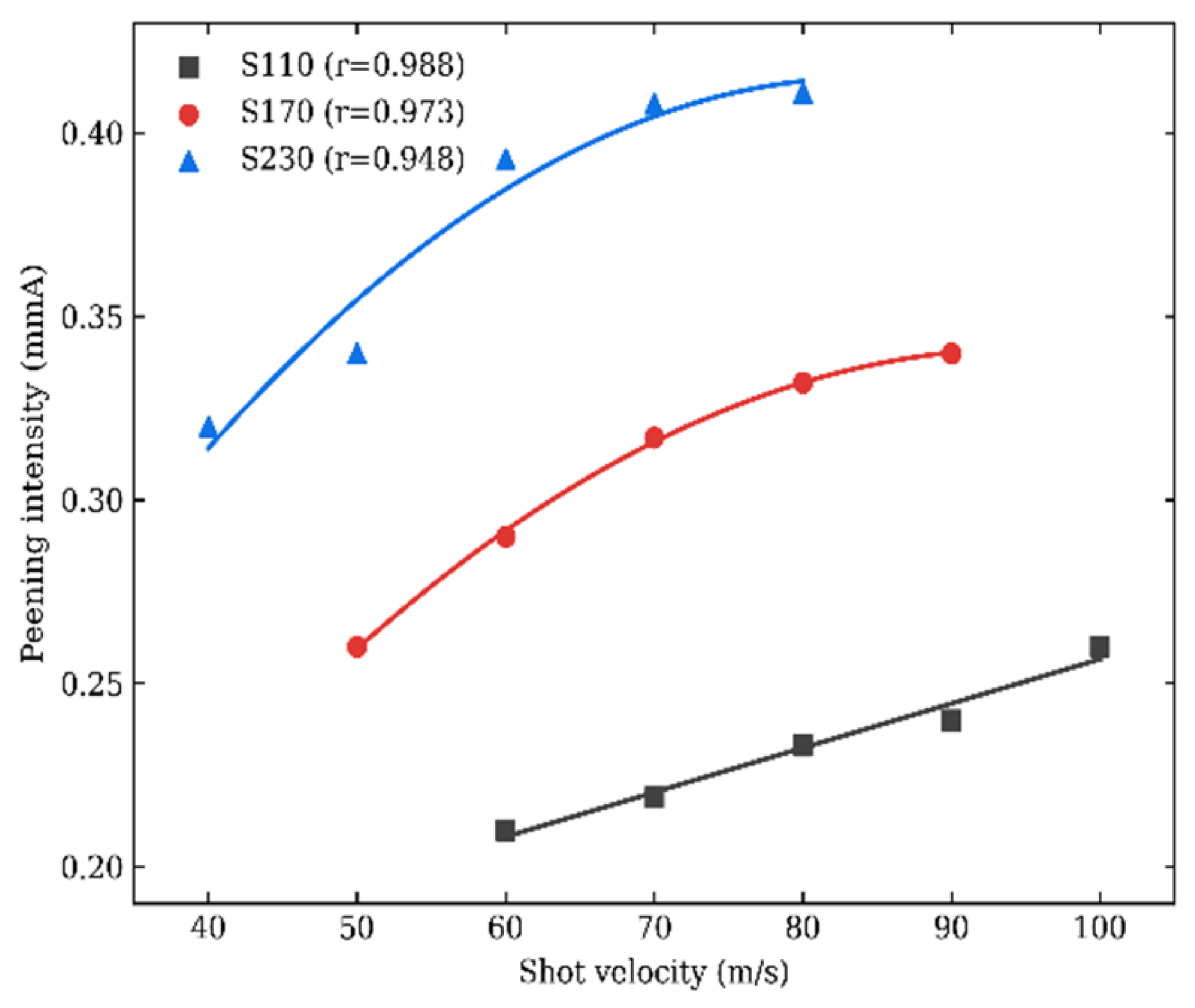
Relationship between simulated shot peening intensity and shot velocity.

Figure 14 shows the effect of shot velocity on shot peening intensity for S110, S170 and S230 high-hardness cast steel shots. For S110 high-hardness cast steel shot, when the shot velocity increased from 60 m/s to 100 m/s, the shot peening intensity increased from 0.205 mmA to 0.262 mmA, representing an increase of 27.8%. For S170 high-hardness cast steel shot, when the shot velocity increased from 50 m/s to 90 m/s, the intensity increased from 0.263 mmA to 0.348 mmA, representing an increase of 32.3%. For S230 high-hardness cast steel shot, when the shot velocity increased from 40 m/s to 80 m/s, the intensity increased from 0.318 mmA to 0.413 mmA. These results indicate that shot peening intensity increases with increasing shot velocity.

**Figure 15:**
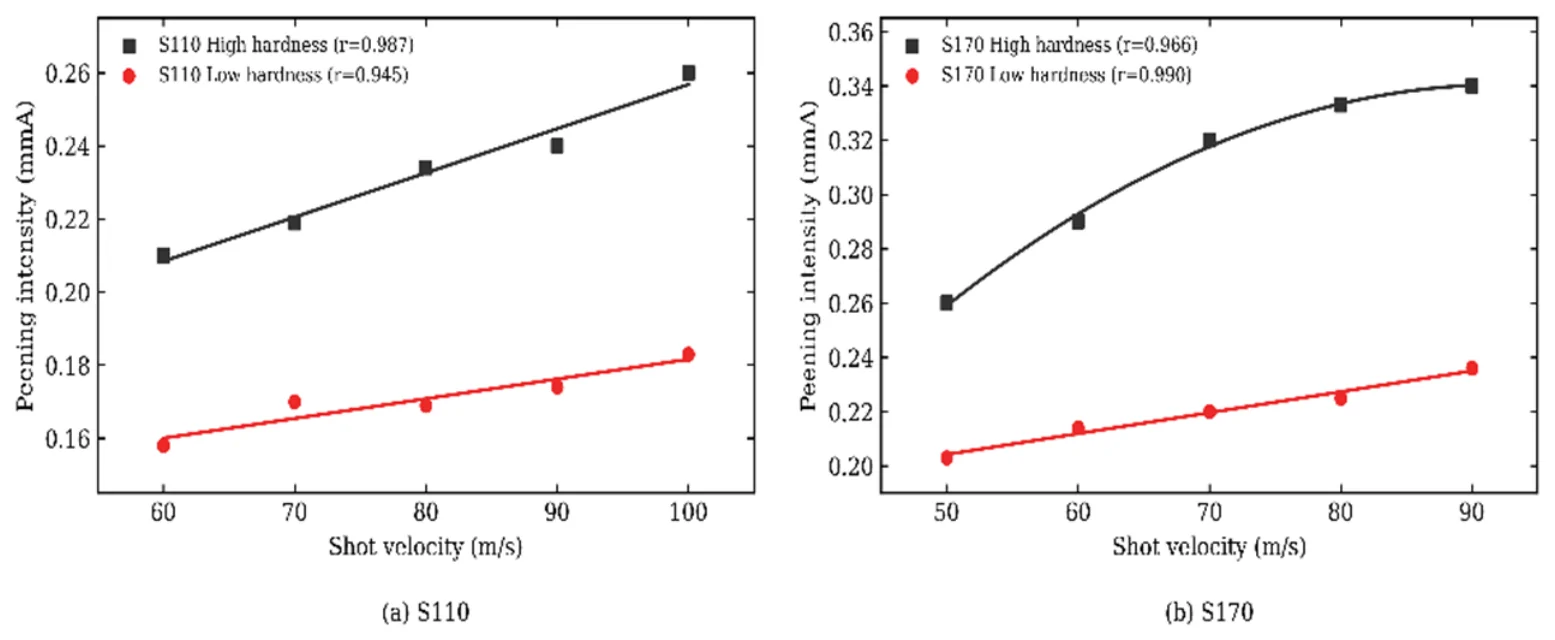
Effect of shot media on shot peening intensity: (**a**) Effect of S110 shot on shot peening intensity; (**b**) Effect of S170 shot on shot peening intensity.

Figure 15 shows the effect of shot hardness on shot peening intensity. At a shot velocity of 60 m/s, the shot peening intensities obtained using S110 high-hardness and low-hardness cast steel shots were 0.205 mmA and 0.156 mmA, respectively. At 100 m/s, the corresponding intensities were 0.262 mmA and 0.183 mmA. At 50 m/s, the intensities obtained using S170 high-hardness and low-hardness cast steel shots were 0.263 mmA and 0.201 mmA, respectively. At 90 m/s, the intensities obtained using S170 high-hardness and low-hardness cast steel shots were 0.348 mmA and 0.234 mmA, respectively. At the same velocity, high-hardness cast steel shots produced significantly higher shot peening intensity than low-hardness cast steel shots.

To quantitatively characterize the relationship between shot velocity and peening intensity, linear and quadratic regression models were fitted to the experimental data in Figure 14 and Figure 15. The corresponding fitting equations, Pearson correlation coefficients, and coefficients of determination were obtained to evaluate the velocity dependence of peening intensity.

As shown in Figure 14, peening intensity increases with shot velocity for all three shot sizes. The S110 shots exhibit an approximately linear relationship, whereas the S170 and S230 shots display nonlinear increasing trends, with a gradual reduction in the rate of increase at higher velocities. The Pearson correlation coefficients are 0.988, 0.973, and 0.948 for S110, S170, and S230, respectively, indicating strong positive correlations between shot velocity and peening intensity.

Similarly, Figure 15 demonstrates that shot hardness significantly affects both the magnitude and velocity dependence of peening intensity. High-hardness shots consistently produce greater peening intensities than low-hardness shots at the same impact velocity. The fitted models exhibit coefficients of determination ranging from 0.892 to 0.997, indicating generally good agreement with the experimental data. These results quantitatively demonstrate the combined influence of shot velocity, size, and hardness on peening intensity.

### 5.2. Effect of Shot Peening Intensity on Residual Stress

**Figure 16:**
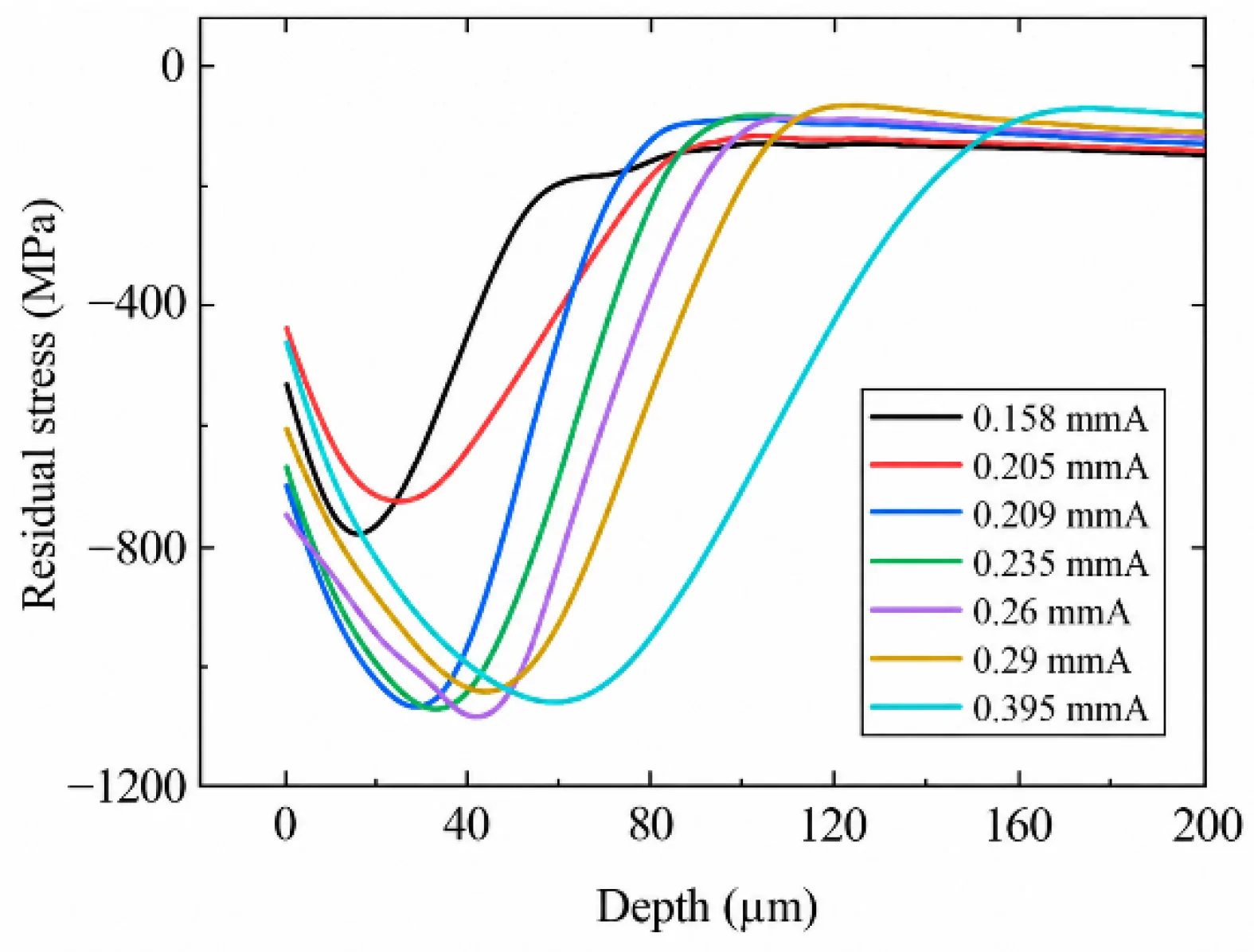
Effect of shot peening intensity on residual stress.

Figure 16 shows the effect of shot peening intensity on residual stress. When the shot peening intensity increased from 0.158 mmA to 0.395 mmA, the position of maximum compressive residual stress increased from 15 μm to 55 μm, representing an increase of 266%, and the compressive residual stress layer depth increased from 50 μm to 160 μm, representing an increase of 220%. The depth of maximum compressive residual stress and the compressive residual stress layer depth were positively correlated with shot peening intensity. However, the surface compressive residual stress and maximum compressive residual stress magnitude did not show a clear trend. This is because shot peening intensity is affected by multiple coupled process parameters; identical shot peening intensities under different parameter combinations may still produce significantly different residual stress distributions.

Almen intensity reflects the macroscopic bending response of a standard Almen strip subjected to shot impacts, whereas residual stress is governed by nonuniform plastic deformation within the material and subsequent elastic recovery during unloading. Although different combinations of shot size, velocity, and hardness may produce similar Almen intensities, they can lead to significant differences in impact kinetic energy, contact pressure, contact area, and plastic deformation depth. Generally, larger shots tend to generate more extensive plastic deformation zones, promoting the development of compressive residual stresses at greater subsurface depths. In contrast, smaller shots impacting at higher velocities may induce more localized near-surface plastic deformation, resulting in steeper plastic strain gradients. Furthermore, differences in strain-rate effects, work hardening, and the accumulation of plastic strain and stress redistribution induced by repeated impacts can further influence the magnitude and depth distribution of residual stresses. Relying solely on Almen intensity is insufficient for precise control of the shot peening process. Almen intensity primarily reflects the macroscopic deformation response of a standard strip and cannot fully characterize the residual stress, surface roughness, and plastic deformation state of the treated component. Even under similar Almen intensities, variations in shot size, velocity, hardness, and coverage can result in significantly different strengthening effects. Therefore, shot peening optimization should comprehensively consider Almen intensity, coverage, shot characteristics, and surface integrity parameters. Establishing quantitative relationships between process parameters and key surface integrity indicators, such as residual stress and surface roughness, is essential for transitioning from intensity-based control to multi-parameter coordinated optimization.

### 5.3. Correlation Between Residual Stress and Shot Peening Intensity in Herringbone Gears

**Figure 17:**
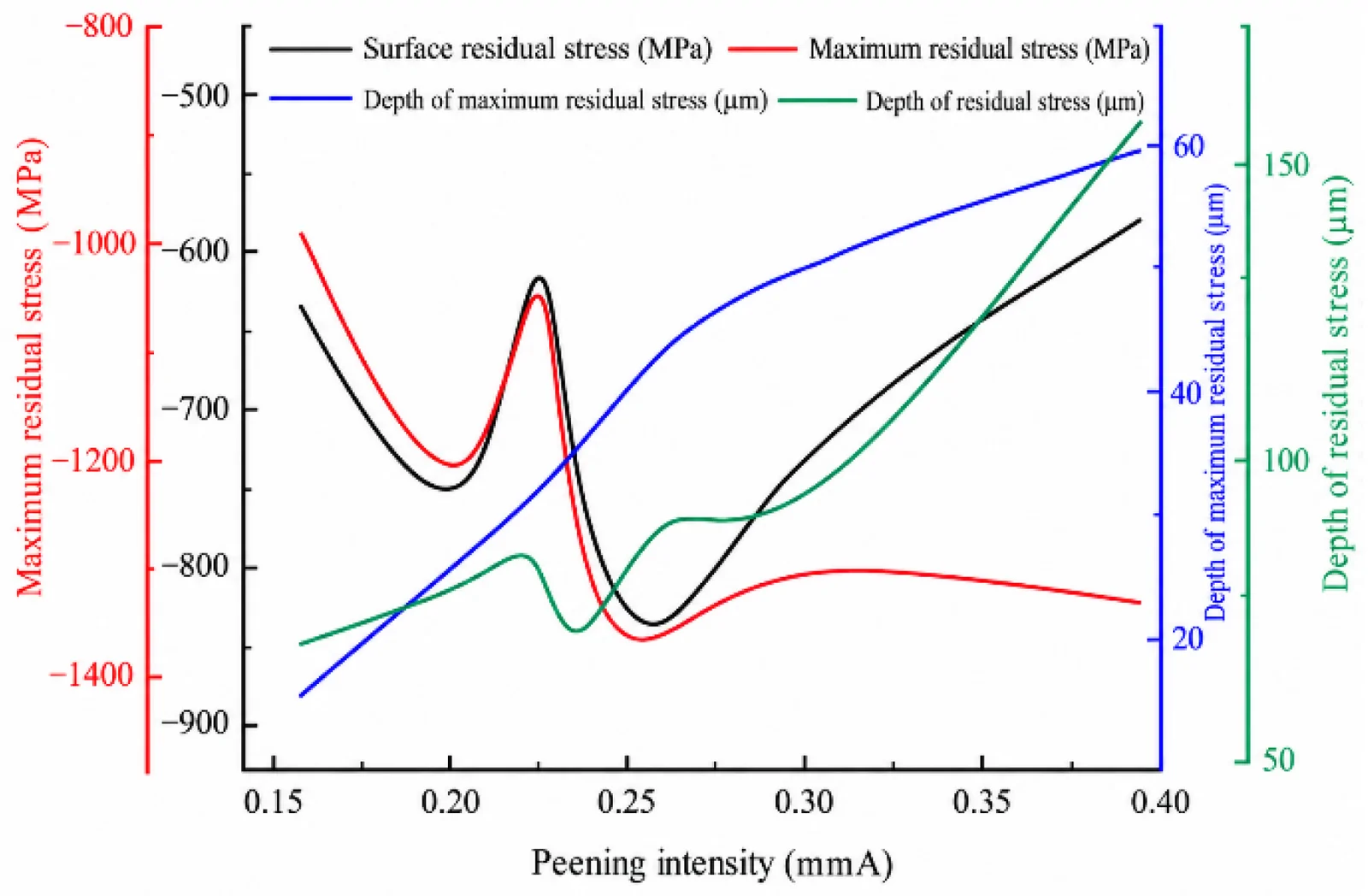
Correlation between shot peening intensity and residual stress in herringbone gears.

After shot peening, the residual stress distribution along the depth direction exhibits a spoon-shaped profile. The maximum compressive residual stress usually appears in the subsurface region; beyond this depth, the compressive residual stress gradually decreases and eventually stabilizes. Surface residual stress, maximum residual stress, the depth of maximum residual stress and residual stress layer depth are key indicators for evaluating residual stress after shot peening. Figure 17 shows the influence of shot peening intensity on these indicators. Within the range investigated in this study, when the shot peening intensity was greater than 0.15 mmA and less than 0.25 mmA, surface residual stress and maximum residual stress showed the same trend. When the shot peening intensity was greater than 0.30 mmA and less than 0.40 mmA, surface residual stress and maximum residual stress showed opposite trends with increasing intensity. The depth of maximum compressive residual stress was always positively correlated with shot peening intensity, and the residual stress layer depth also generally increased with intensity. In summary, the influence of shot peening intensity on residual stress layer depth and maximum residual stress depth is relatively clear because an increase in intensity represents an increase in impact energy, which inevitably produces a deeper strengthened layer. In contrast, the influence of shot peening intensity on surface residual stress and maximum residual stress magnitude is ambiguous because intensity is governed by multiple coupled process parameters, and different residual stress states may be obtained at the same intensity.

## 6. Conclusions

This study investigated the factors influencing shot peening intensity and the effect of shot peening intensity on residual stress. The following conclusions can be drawn:

(1) Under the conditions investigated in this study, when S110 high-hardness cast steel shot was used and the shot velocity increased from 60 m/s to 100 m/s, the shot peening intensity increased from 0.205 mmA to 0.262 mmA. At a shot velocity of 60 m/s, the intensities obtained using S110, S170 and S230 high-hardness cast steel shots were 0.205 mmA, 0.283 mmA and 0.385 mmA, respectively. At the same velocity, the intensities produced by S110 high-hardness and low-hardness cast steel shots were 0.205 mmA and 0.156 mmA, respectively.
(2) Shot peening intensity is positively correlated with shot velocity, shot diameter and shot hardness. Because it is affected by coupled process parameters, identical shot peening intensities may still lead to different residual stress states.
(3) Under the conditions investigated in this study, surface residual stress and maximum residual stress did not show an obvious monotonic variation with shot peening intensity. The residual stress layer depth and the depth of maximum compressive residual stress were positively correlated with shot peening intensity. When the intensity increased from 0.158 mmA to 0.395 mmA, the position of maximum compressive residual stress increased from 15 μm to 55 μm, representing an increase of 266%, and the compressive residual stress layer depth increased from 50 μm to 160 μm, representing an increase of 220%.

## Figures and Tables

**Table 1:** Material parameters of the target and shot models.

Material Parameter	HRC	Yield Strength (MPa)	Mean Shot Diameter (mm)
S110 high-hardness cast steel shot	55~62	1838	0.35
S110 low-hardness cast steel shot	45~52	1350	0.35
S170 high-hardness cast steel shot	55~62	1838	0.50
S170 low-hardness cast steel shot	45~52	1350	0.50
S230 high-hardness cast steel shot	55~62	1838	0.70

**Table 2:** Process parameters for Almen strip shot peening.

Process Parameter	Value
Peening angle	90°
Shot mass flow rate	6 kg/min
Peening air pressure	2~5 bar
Stand-off distance	150 mm
Shot type	S110 high-hardness cast steel shot

**Table 3:** Material parameters of the DEM model for herringbone gear shot peening.

Gear Material Parameter	Value	Shot Material Parameter	Value
Elastic modulus *E_g_* (GPa)	210	Elastic modulus *E_s_* (GPa)	210
Density *ρ_g_* (kg/m3)	7800	Density *ρ_s_* (kg/m3)	7800
Poisson’s ratio *ν_g_*	0.3	Poisson’s ratio *ν_s_*	0.3

**Table 4:** Chemical composition of 10CrNi2Mo3Cu2V steel (%) [1].

Cr	Ni	Mo	Cu	V	C	Mn	Si	Fe
1.0	2.0	3.25	2.0	0.1	0.11	0.4	0.9	Balance

**Table 5:** J-C constitutive model parameters of 10CrNi2Mo3Cu2V steel [1].

*ε*_0_ (s^−1^)	*A* (MPa)	*B* (MPa)	*n*	*C*	*m*
1000	1900	1523	0.2269	0.0613	0.945

## Data Availability

The data presented in this study are available on request from the corresponding author.
